# Influence of Cation Chain Length and Anion Identity in Imidazolium-Based Ionic Liquids on Lignin Depolymerization via Acid Hydrolysis

**DOI:** 10.3390/molecules31101609

**Published:** 2026-05-11

**Authors:** Diana Lucía Campa-Guevara, Nohra Violeta Gallardo-Rivas, Erick Orlando Merinos-Herrera, Fausto Antonio Balderas-Jaramillo, Erika Alarcón-Ruiz, Luis Daniel Ordóñez-Pacheco

**Affiliations:** Tecnológico Nacional de México—I. T. Cd. Madero, Los Mangos, 1 de Mayo s/n, Ciudad Madero 89440, Tamaulipas, Mexico; diana.cg@cdmadero.tecnm.mx (D.L.C.-G.); nohra.gr@cdmadero.tecnm.mx (N.V.G.-R.); erick.mh@cdmadero.tecnm.mx (E.O.M.-H.); fausto.bj@cdmadero.tecnm.mx (F.A.B.-J.)

**Keywords:** lignin, ionic liquids, imidazolium-based ionic liquids, depolymerization, acid hydrolysis, biomass valorization

## Abstract

In this work, the ability of a series of imidazolium-based ionic liquids (ILs) to depolymerize Kraft lignin through acid hydrolysis was evaluated. ILs featuring two-, four-, and six-carbon alkyl chains combined with [Cl^−^], [BF_4_^−^] and [CH_3_COOH^−^] anions were studied to determine the influence of cation and anion structure. The twelve ILs were synthesized and characterized by FT-IR and ^1^H/^13^C NMR spectroscopy. Results indicate that both the anion and cation significantly affect depolymerization efficiency; specifically, longer alkyl chain lengths correlated with higher conversion percentages. Anion efficacy followed the order: [Cl^−^] > [CH_3_COOH^−^] > [BF_4_^−^]. Furthermore, reaction temperature did not show a significant impact on conversion within the studied range. Spectroscopic data suggest that bond dissociation follows a Brønsted acid-catalyzed mechanism, evidenced by the reduction of phenolic components and guaiacyl/syringyl units in the recovered lignin samples.

## 1. Introduction

Growing environmental awareness, coupled with the uncertain future of fossil fuels, has sparked growing interest in the search for renewable alternatives, like solar energy, eolic energy and biomass, to satisfy the energy needs of the world.

Biomass is any organic matter that is renewable with due time, and among the diversity of renewable resources, it is the only renewable carbon source in nature, providing a unique advantage for the production of value-added products, as well as being the fourth energy source, after petroleum, carbon and natural gas [1,2]. In the last 10 years, the interest in the conversion of biomass into chemical products has strongly increased within industrial companies and academic institutions, due to the global energy crisis and its relation to environmental impact. In industry, biomass is primarily utilized to generate heat and power, but it can also be processed into value-added chemical products and high-quality fuels [3,4]. Consequently, the conversion of biomass into these valuable resources can help mitigate the heavy reliance on fossil fuels and reduce environmental carbon dioxide (CO_2_) emissions. The most important sources of biomass include forest waste products, agricultural waste, energy crops, aquatic plants, organic industrial waste and green residues, which need pre-treatment by way of chemical, physical or biological mediums to break the chemical bonds present in the structure of the biomass [1,5]. Lignocellulosic biomass is the most abundant form of biomass on Earth, with an annual production of around 170 billion metric tons. It is comprised of three main components: cellulose (40–50%), a crystalline glucose polymer; hemicellulose (20–30%), a complex amorphous polymer, whose main component is the manometric unit xylose; and lignin (15–40%), a complex amorphous tridimensional polymer consisting of methoxylated phenylpropanoid units [1,2,6].

As the second most abundant natural biopolymer, after cellulose, lignin has caught the attention of many scientists for a long time, due to its complexity, lack of uniformity and its bonds to other substances [7]. The term lignin is used to define an irregular polyphenolic amorphous biopolymer, the biological function of which is, along with polysaccharides, to generate structures of resistant vegetable tissue, granting great mechanical resistance to the plants, a reason for which it is considered the natural glue of plants [8,9]. Lignin is generated via the oxidative polymerization of three phenolic precursors, phenylpropanes or monolignols, differing in the grade of substitution of the methoxy group, -OCH_3_, in the phenolic ring: ρ-coumaric alcohol (without methoxyl), coniferyl alcohol (one methoxy group) and sinapyl alcohol (two methoxy groups).

**Figure 1:**
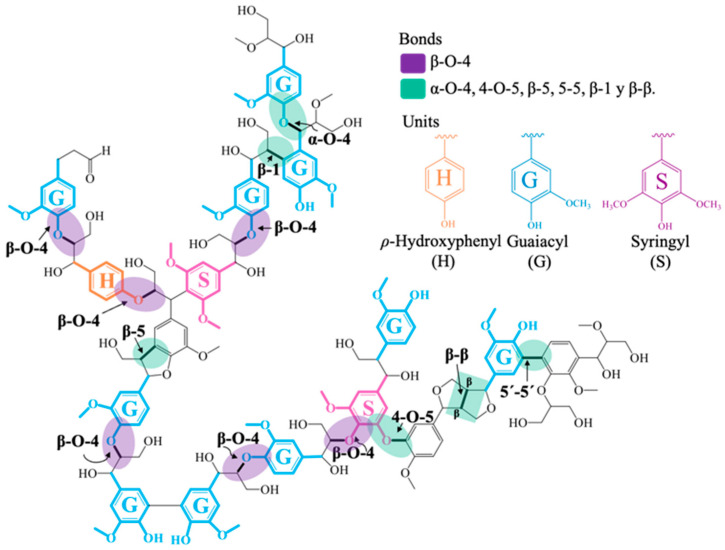
Representative diagram of the structure of the lignin molecule, adapted from Ref. [11].

These monolignol units, when incorporated into the lignin polymer, create a heterogeneous composite of phenylpropanoid units generically denominated as: ρ-hydroxyphenyl (4-hidroxyphenyl, H), guaiacyl (4-hidroxy-3-methoxyphenyl), and syringyl (4-hidroxy-3,5-dimethoxiphenyl, S) [9,10]. In the lignin polymer’s matrix, the monolignol fractions are in linked through a variety of interunit bonds of alkyl- or aryl-ether (∼60–70%), carbon–carbon (∼25–35%) and very low ester bonds (<5% in herbaceous plants) which include β-O-4, β-5, β-β, β-1, β-6, α-β, α-O-4, α-O-γ, γ-O-γ, 1-O-4, 4-O-5, 1-5, 5-5 and 6-5. According to the literature, the seven main linkages in lignin are: β-aryl-ether (β-O-4), α- aryl-ether (α-O-4), biphenyl-ether (4-O-5), phenylcoumaran ((β-5), biphenyl (5-5), 1,2-diarylpropane (β-1) and resinol (β-β/α-O-5), Figure 1 [11].

The proportion of bonds in the biopolymer structure depends on not only the type of wood but on the part of the plant as well. However, the most predominant bond in the lignin polymer belongs to the ether β-O-4 type, also called acylglycerol-β-aryl, which represents around 40–60% of all the different types of bonds found in the structure. As such, the lignin macromolecule has very little free hydroxyl phenolic molecules because the majority of these participate in the ether bonds present in the monomeric units, fundamentally with the central carbon of the chain lateral to the propyl of the other unit and with benzylic carbons and, to a lesser extent, with terminal carbons, which is why it is these phenolic and aryl-ether bonds that cleave during any delignification process. The structural model of the native lignin suggests that this material could be converted into aromatic compounds, phenols and saturated cyclicals, and, depending on the type of lignin, this can be converted into many products. An example is Kraft Lignin, which is used currently as a fuel in the Kraft pulp process and can also be used in the production of carbon and composite fibers. Lignosulfonates have also been proposed as adhesives, concrete plastificants and colorant dispersers. Additionally, lignin has been used in polymeric applications as a stabilizer, tensoactives, epoxy resins and superabsorbent hydrogels [8,12,13]. Besides the aforementioned applications, the depolymerization of lignin into its aromatic components results in an alternative for the petrochemical industry and, maybe, could be the most promising way to sustainably use lignin.

The processes for depolymerization, focused on the dissociation of the weak ether bonds α-O-4 and β-O-4, are the most favorable strategy enthalpically. Due to the high molecular weight of lignin, the first step in depolymerization usually involves the selective rupture of the macromolecule of the polymer into small fragments. Among the most exploited strategies are pyrolysis, catalytic hydrogenation, oxidation, depolymerization catalyzed by an acid or a base, hydrodisintegration, biodegradation, among others, by which many products such as phenols, aromatic compounds like benzene, toluene, xylene, quinones, cyclohexenes, methane, ethane, propane and olefins can be obtained.

However, the percentage of conversion of the depolymerization of lignin to monomeric molecules is low [14]. The above is due in large part to the complexity of the structure of the biopolymer, which constitutes a great obstacle for its chemical valorization. The recalcitrant structure of lignin results in low solubility in conventional organic solvents, which is why the technologies used currently in the isolation or processing of lignin employ high temperatures (between 250 °C and 650 °C) and/or rough alkaline or acidic conditions. These conditions usually give rise to irreversible condensations through electrophilic substitutions in aromatic nuclei rich in electrons that result in a complex mixture of alkylated phenolic and polyhydroxylated compounds, as well as volatile compounds and carbon, which presents challenges in the post-process to separate phenolic-type compounds or to obtain better, more homogeneous mixtures [2,15,16].

In some cases, like pyrolysis, hydrocracking and depolymerization catalyzed by an acid and/or a base, the small fragments of lignin obtained need a second step to eliminate, maintain, or regenerate functional groups present in the primary products and obtain specific products. The exploration and development of strategies of depolymerization that are profitable, as well as the use of new catalysts and/or solvents for the valorization of lignin, have been of vital importance in the last decade [17]. In this context, recent research has aimed to enhance biorefinery sustainability by developing ultrafast pretreatment strategies, such as the use of microwave-responsive deep eutectic solvents (DES), which can achieve high lignin removal at low severity factors [18]. While these emergent technologies offer significant environmental and economic benefits by reducing reaction times, it remains essential to systematically understand how the specific chemical structure of traditional designer solvents, such as imidazolium-based ionic liquids (ILs), influences bond cleavage under conventional acid hydrolysis.

The present work is focused on the depolymerization catalyzed by acid, DCA, also known as acid hydrolysis, which has been studied and applied for a long time for the depolymerization and generation of simple aromatic monomers from lignin in relatively soft conditions. Currently, most of the reported work in this field has been centered upon the hydrolytic rupture of predominantly aryl- alkyl-ether bond types (for example, β-O-4 bonds), because these bonds are the weakest in regard to the structure of lignin in comparison to others, like aryl-aryl-ether, C-O phenolic and -C-C bonds, among aromatic remains. Historically, DCA has been primarily used for the isolation of lignin fragments from the lignocellulose matrix, rather than for the depolymerization of lignin into valuable aromatic monomers. The first lignin hydrolysis reaction catalyzed by acid was brought to attention in 1924, when Hägglund and Björkman distilled lignin with hydrochloric acid at 12% to obtain thiobarbiturate acid, phloroglucinol and barbituric acid. Throughout the years, different types of acids, including mineral acids, Lewis acids, acidic zeolites, and organic acids, as well as the integration of ILs as a solvent and catalyst, have been tested in the depolymerization of lignin and its model compounds veratrylglycerol-β-guaiacyl l ether (VG) and guaiacylglycerol--β-guaiacyl ether (GG). In general, the final reaction finalizes in the first two to four hours of reaction, severe reactions are required, with temperature ranges between 140 °C and 400 °C, and the yields are low [15,19].

The term IL is used to describe a class of organic salts that are liquid at temperatures below °C; they are characterized by possessing an ample number of physicochemical properties, such as low vapor pressure, non-flammability, chemical stability at high temperatures and miscibility with polar and non-polar solvents [20,21,22]. ILs have emerged recently as popular solvents to dissolve lignocellulose and its main components. It has been proven that ILs are advantageous solvents for the conversion of lignin due to their ability to break β-O-4 bonds under low temperatures (temperatures below 250 °C) and dissolve them easily, as well as their favoring reactions that form carbocations [23,24].

Recent investigations about the use of ILs for the dissolution of lignin and model compounds focus on the study of reactions used for conversion into useful chemical products, reducing operating costs and increasing yield and selectivity. In 2008, Kubo et al. [25,26] observed and studied in great detail the conversion of the model compound of lignin GG using dialkylimidazole-base ILs [BMIM^+^][Cl^−^], [AMIM^+^][Cl^−^] and [EMIM^+^][CH_3_COOH^−^] and heat-obtaining as a main product enol-ether, glycerol 3-(4hydroxy-3-methoxypehenyl)-2-(methoxy phenoxy)-2-propenol (EE) stable intermediate. Driven by this discovery, in 2010, Stärk et al. [27] attempted the depolymerization of organosolv lignin from beech trees in the presence of IL trifluoromethanesulfonate of 1-ethyl-3- methylimidazolium [EMIM^+^][CF_3_SO_3_^−^] associated with magnesium nitrate, Mn(NO_3_)_2_, catalyzing at 100 °C and 8.4 Mpa, successfully separating pure 2,6-dimethoxy-1,4-benzoquinine (DMBQ) with a 11.5% yield. In that same year, Binder et al. [19,28] used heterogeneous and homogeneous catalysts for the conversion of model compounds of lignin eugenol (2-methoxy-4-(2-propenyl) phenol, 4-ethyl-2-,methoxypenhyl and 2-phenylethylphenylether using imidazolium-based ILs as a solvent, proving that ionic liquids not associated with acids, with the right catalyst, can result in processes with high percentages of selectivity and conversion. Also, in 2010, a group of researchers led by Ekerdt y Jia [25,29] studied the acid hydrolysis of model compounds of phenol GG base lignin and anisol base (VG) using the Brønsted acid IL chloride 1-H-3-methylimidazolium [hMIM^+^][Cl^−^] as a catalyst and a solvent, and found that more than 70% of the β-O-4 of GG and VG bonds reacted with water to produce guaiacyl at 150 °C.

A year later, the hydrolysis of the same lignin model compounds, GG and VG, using the IL [BMIM^+^][Cl^−^] in the presence of water and metallic chlorides was reported; the Lewis acid/H_2_O/IL catalytic system using FeCl_3_, AlCl_3_ and CuCl_2_ resulted in high effectiveness for the cleavage of β-O-4 linkages in the GG model, achieving 100% conversion at 150 °C to obtain 80% guaiacyl as the final product. The VG model was more resistant to degradation, with approximately 75% of the β-O-4 linkages reacting under the same conditions; in addition, Hibbert’s ketones were formed due to repolymerization and condensation [25,30].

After, in 2012, Cox et al. [25,31] tested the depolymerization of lignin derived from oak (extracted with [EMIM][CH_3_COOH^−^]) with the ionic liquid [hMIM+][Cl^−^] and proved, with NMR and IR, that lignin depolymerized through hydrolysis and alkyl-aryl ether bonds. Thanks to these studies, it was determined that hydrolysis of lignin and its model compounds in ionic liquids follows the reaction mechanism of the reaction catalyzed by Brønsted Acid [16]. As demonstrated, ionic liquids (ILs) possess a significant capacity to enhance the yields of reactions aimed at lignin depolymerization and its subsequent conversion. Due to the tunable design properties of ILs, the challenge lies in identifying specific ILs that maximize these results, enabling the production of high-value-added products from this renewable resource.

The depolymerization of lignin by hydrolysis into intermediary products more susceptible to chemical or biological transformation is the first step in producing biofuel and value-added chemicals. Acid hydrolysis is typically performed using either dilute acid solutions at high temperatures or concentrated acids at low temperatures. Over the years, various treatments have been investigated to optimize hydrolysis yields by adjusting variables such as reaction time, temperature, and acid concentration. Generally, conventional methods utilize temperature ranges of 80–200 °C and reaction times between 40 and 274 min [32]. More recently, ILs have been employed as both reaction media and catalysts to enhance the yields achieved through traditional hydrolysis methods [33].

## 2. Results and Discussion

### 2.1. Synthesis of Ionic Liquids

In this work, an acid-assisted depolymerization was carried out by ILs derived from imidazole, whose use has been reported in the treatment and dissolution of lignocellulosic biomass, lignin and model compounds of lignin [31,32,33,34,35]. To evaluate the effect of the length of the alkyl substituent in the imidazolium ring, rings of two, four and six carbons were taken into consideration. To evaluate the effect of the anion on the properties of IL, cations were combined with [Cl^−^], [BF_4_^−^] and [CH_3_COOH^−^], which have been reported in the industrial pretreatment of lignocellulose, as seen in Table 1 [36,37,38].

The synthesis of ionic liquids was done in two steps. The first step’s main goal was to form the anion–cation ionic pair via the alkylation reaction of 1-methylimidazolium with the halide of the corresponding alkyl to obtain an IL precursor. The second step involved reactions for an ionic exchange or metathesis to incorporate anions of interest into the system. Having concluded the synthesis, the ILs were spectroscopically characterized through nuclear magnetic resonance (NMR) of ^1^H and ^13^C, and infrared spectroscopy (FT-IR). The results of the characterization are the following: the chemical displacements (δ) were reported in parts per million (ppm) using as internal references the chemical displacement of tetramethylsilane for the experiments of ^1^H and ^13^C NMR. The constant values of coupling (J) are in hertz (Hz). The multiplicity of signals was denoted with the following abbreviations: s for simple signals, d for double signals, t for triple signals and m for multiple signals.

(a) 1-ethyl-3-methylimidazolium chloride: ^1^H NMR (400 MHz, D2O) δ (ppm): 8.65 (s, 1H), 7.42 (s, 1H), 7.36 (s, 1H), 4.16 (c, J = 7.3 Hz, 2H), 3.82 (s, 3H), 1.43 (t, J = 7.4 Hz, 3H). ^13^C NMR (100 MHz, D2O) δ (ppm): 135.57, 123.46, 121.89, 44.81, 35.67, 14.55. IR (neat) ν (cm^−1^): 3376, 3049, 2978, 2864, 2745, 1572, 1458, 1384, 1333, 1170, 1021, 888, 793, 698, 627.

(b) 1-ethyl-3-methylimidazolium tetrafluoroborate: ^1^H NMR (400 MHz, D2O) δ (ppm): 8.63 (s, 1H), 7.45 (s, 1H), 7.38 (s, 1H), 4.20 (c, J = 7.3 Hz, 2H), 3.86 (s, 3H), 1.47 (t, J = 7.3 Hz, 3H). ^13^C NMR (100 MHz, D2O) δ (ppm): 135.60, 123.48, 121.87, 44.80, 35.57, 14.45. IR (neat) ν (cm^−1^): 3164, 3121, 1572, 1456, 1337, 1286, 1171, 846, 754, 623, 521.

(c) 1-ethyl-3-methylimidazolium acetate: ^1^H NMR (400 MHz, D2O) δ (ppm): 8.59 (s, 1H), 7.37 (s, 1H), 7.30 (s, 1H), 4.10 (c, J = 7.3 Hz, 2H), 3.77 (s, 3H), 1.79 (s, 3H), 1.37 (t, J = 7.3 Hz, 3H). ^13^C NMR (100 MHz, D2O) δ (ppm): 180.64, 135.52, 123.41, 121.83, 44.73, 35.55, 23.01, 14.45. IR (neat) ν (cm^−1^): 2976, 1566, 1378, 1175, 1089, 1000, 898, 803, 634.

(d) 1-butyl-3-methylimidazolium chloride: ^1^H NMR (400 MHz, D2O) δ (ppm): 8.65 (s, 1H), 7.41 (s, 1H), 7.36 (s, 1H), 4.12 (t, J= 7.1 Hz, 2H), 3.82 (s, 3H), 1.77 (q, J = 7.3 Hz, 2H), 1.24 (m, 2H), 0.84 (t, J = 7.4 Hz, 3H). ^13^C NMR (100 MHz, D2O) δ (ppm): 137.47, 123.71, 122.04, 49.59, 36.41, 32.05, 19.32, 13.33. IR (neat) ν (cm^−1^): 3384, 3056, 2959, 2873, 1567, 1462, 1379, 1169, 1021, 870, 754, 622, 516.

(e) 1-butyl-3-methylimidazolium tetrafluoroborate: ^1^H NMR (400 MHz, D2O) δ (ppm): 8.60 (s, 1H), 7.40 (s, 1H), 7.35 (s, 1H), 4.12 (t, J = 7.1 Hz, 2H), 3.82 (s, 3H), 1.78 (q, J = 7.2 Hz, 2H), 1.25 (m, 2H), 0.85 (t, J = 7.3 Hz, 3H). ^13^C NMR (100 MHz, D2O) δ (ppm): 135.82, 123.47, 122.19, 49.27, 35.58, 31.28, 18.76, 12.61. IR (neat) ν (cm^−1^): 3031, 2959, 2934, 2866, 1575, 1463, 1379, 1175, 1001, 899, 754, 622, 632.

(f) 1-butyl-3-methylimidazolium acetate: ^1^H NMR (400 MHz, D2O) δ (ppm): 8.60 (s, 1H), 7.37 (s, 1H), 7.32 (s, 1H), 4.08 (t, J = 7.1 Hz, 2H), 3.78 (s, 3H), 1.79 (s, 3H), 1.73 (q, 2H), 1.20 (m, 2H), 0.81 (t, J = 7.3 Hz, 3H). ^13^C NMR (100 MHz, D2O) δ (ppm): 181.17, 135.81, 123.44, 122.18, 49.24, 35.57, 31.24, 23.28, 18.72, 12.60. IR (neat) ν (cm^−1^): 3163, 3121, 2964, 2937, 2878, 1574, 1466, 1385, 1288, 1170, 1048, 847, 1048, 847, 753, 623, 520.

(g) 1-hexyl-3-methylimidazolium chloride: ^1^H NMR (400 MHz, D2O) δ (ppm): 8.62 (s, 1H), 7.39 (s, 1H), 7.34 (s, 1H), 4.11 (t, J = 7.1 Hz, 2H), 3.81 (s, 3H), 1.78 (m, 2H), 1.21 (m, 6H), 0.77 (t, J = 6.0 Hz, 3H). ^13^C NMR (100 MHz, D2O) δ (ppm): 135.80, 123.47, 122.21, 49.58, 35.66, 30.32, 29.15, 24.98, 21.77, 13.23. IR (neat) ν (cm^−1^): 3384, 3056, 2929, 2856, 1568, 1470, 1379, 1168, 1022, 870, 763, 623.

(h) 1-hexyl-3-methylimidazolium tetrafluoroborate: ^1^H NMR (400 MHz, D2O) δ (ppm): 8.60 (s, 1H), 7.39 (s, 1H), 7.34 (s, 1H), 4.11 (t, J = 7.1 Hz, 2H), 3.81 (s, 3H), 1.78 (m, 2H), 1.22 (m, 6H), 0.77 (s, 3H). ^13^C NMR (100 MHz, D2O) δ (ppm): 135.79, 123.47, 122.19, 49.46, 35.59, 30.26, 29.14, 24.97, 21.76. IR (neat) ν (cm^−1^): 3160, 2932, 2861, 1709, 1573, 1462, 1364, 1286, 1169, 1050, 850, 761, 623, 520.

(i) 1-hexyl-3-methylimidazolium acetate: ^1^H NMR (400 MHz, D2O) δ (ppm): 8.66 (s, 1H), 7.42 (s, 1H), 7.37 (s, 1H), 4.12 (t, J = 7.0 Hz, 2H), 3.82 (s, 3H), 1.86 (s, 3H), 1.79 (m, 2H), 1.21 (m, 6H), 0.76 (s, 3H). ^13^C NMR (100 MHz, D2O) δ (ppm): 180.09, 135.82, 123.53, 122.26, 49.61, 35.75, 30.38, 29.22, 25.04, 22.75, 21.82, 13.31. IR (neat) ν (cm^−1^): 3072, 2931, 2956, 2860, 1567, 1460, 1393, 1276, 1167, 1006, 865, 762, 621.

### 2.2. Acid Hydrolysis of Lignin Assisted with ILs

Once the acid hydrolysis of lignin, assisted with the desired ionic liquids, was finished, the lignin was dried and weighed to obtain the yields obtained in each reaction. Changes were observed in coloration and physical conformation in the lignin samples obtained after drying, in contrast with the dark brown coloration and fine consistency of the original Kraft lignin. To obtain the conversion percentages of lignin during the acid hydrolysis assisted with ionic liquids, we used Equation (1).

(1) $$Conversion\;\left(\%\right)=\frac{grams\;of\;lignin-grams\;of\;dry\;lignin}{grams\;of\;lignin}*100,$$

where the grams of lignin correspond to the grams of Kraft lignin added to carry out the hydrolysis reaction and that satisfied the relation, 1:10 lignin:IL, and the grams of dry lignin correspond to the grams of lignin obtained after the drying of the solid residue obtained during the depolymerization reaction; this fact was obtained subtracting the weight of the filter paper with dry lignin and the weight of the clean and dry filter paper before filtering the lignin of the mixture of the reaction. The conversion percentages for the series of imidazolium-based ionic liquids, whose chemical structures and nomenclature are defined in Table 1, are summarized in Table 2.

In the conversion percentages of lignin, for both temperatures, an effect of the ion was observed in the efficiency of the ionic liquid to depolymerize the Kraft lignin, the tendency that determines the efficiency of IL is: [Cl^−^] > [CH_3_COOH^−^] > [BF_4_^−^] and the anion with the least ease to depolymerize lignin is the ion [BF_4_^−^], which is congruent with the results obtained by Cox et al. [39], where the effect of coordination is capable of destabilizing and generating intermediary products that facilitate the dissociation of ether type bonds and result in the liberation of guaiacyl, for which ILs containing anions that are less coordinated like the ion [BF_4_^−^] form significantly higher amounts of vinyl ether (VE9 instead of EE), reducing their capacity to depolymerize lignin. The anions play an important role when forming hydrogen-bond-type interactions, which is why the higher percentages of efficiency present in the ion [Cl^−^] are likely thanks to the ILs, with said ion being capable of more easily breaking and forming interactions of this chemical nature. Although it would be expected that the ion [CH_3_COOH^−^] would have a higher capability than the [Cl^−^] ion to form hydrogen bonds with the hydroxyl groups of lignin (because they have two negatively charged atoms of oxygen) and as a consequence of, result in higher conversion percentages than obtained with the [Cl^−^] ion, it is possible that the steric impediment of the [CH_3_COOH^−^] ion impedes its penetration of the molecular structure of lignin and the formation of stabilizing hydrogen bonds necessary to decompose it in the same magnitude as the ILs with a [Cl^−^] ion. Furthermore, there is an effect of the chain length of the substituent alkyl of the imidazolium cation on the efficiency percentages of the acid depolymerization process of lignin assisted by IL because it is higher with this one than with the ion [HMIM^+^] > [BMIM^+^] > [EMIM^+^], which is consistent with Balaji et al. [40], whose theoretic results found that a higher cation size promoted the dissolution of lignin because it has a larger van der Waals area, and thanks to this greater area of interaction, the cation can more easily break these types of interactions like hydrogen bonds and the natural π–π stacking of lignin to form new and stronger interactions, of the same chemical nature, with the imidazolium ring and alkyl substituent. Finally, a slight increase in lignin conversion percentages was observed during the IL-assisted acid depolymerization process as the reaction temperature increased; however, the established trends regarding the effects of the cation and anion remained constant.

### 2.3. Spectroscopic Characterization of Lignin with Infrared Spectroscopy

**Figure 2:**
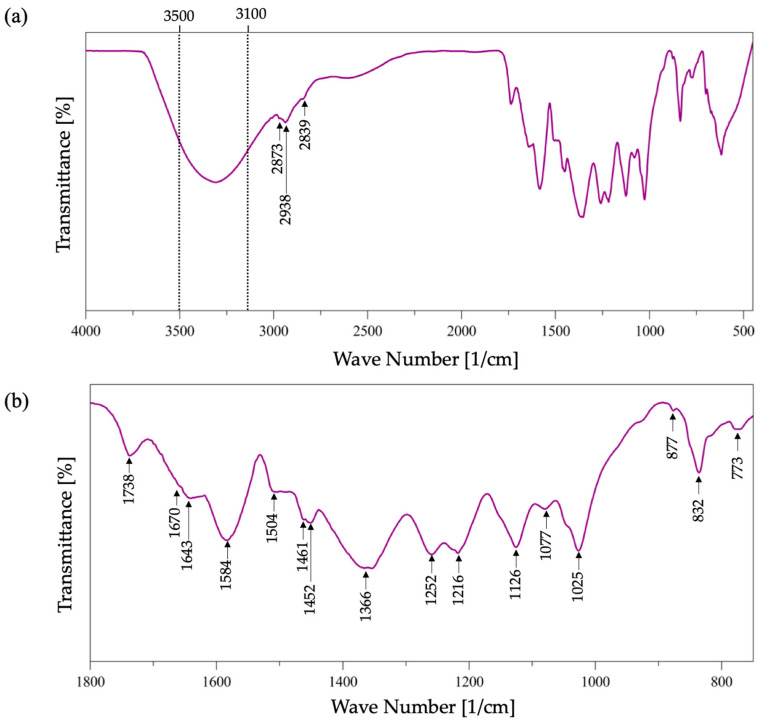
(**a**) Infrared spectrum of Kraft lignin; (**b**) amplification of the spectral region from 1800 to 750 cm^−1^ from the infrared spectrum of Kraft lignin.

The lignin and Kraft lignin obtained during the treatment of acid hydrolysis with ILs in the study were analyzed to evaluate the structural changes obtained in the applied depolymerization process. Thereafter, the obtained results will be presented; firstly, the characterization of Kraft lignin will be explained in detail, followed by the observed changes for every family of IL used in this work. In Figure 2a, the infrared spectrum of Kraft lignin is shown, with an amplification of the spectral region that covers 1800 and 800 cm^−1^ presented in Figure 2b. In the spectrum, it is observed that Kraft lignin shows an ample band between 3500 and 3100 cm^−1^, attributable to the OH stretching of the phenolic and aliphatic groups; the peaks at 2973 and 2983 cm^−1^ of the contracted band of this zone in the spectrum correspond to the stretching of the C-H of the methyl group (CH_3_) and methylene (CH_2_). In addition, the peak at 2893 cm^−1^ corresponds to the stretching of the C-H from group O-CH_3_: the weak band at 1738 cm^−1^ is attributed to the stretching of the non-conjugated carbonyl group C=O with a small arm at 1670 cm^−1^ that is associated with the C=O conjugated stretching of the coniferyl and sinapyl aldehydes. Subsequently, the aromatic skeletal vibrations in combination with C-H stretching in the region between 1643 and 1452 cm^−1^ correspond to the characteristic peaks of Kraft lignin; 1643 cm^−1^ corresponds to the C=C stretch of the conjugated aromatic ring of the coniferyl and sinapyl alcohols; the 1584 and 1504 cm^−1^ peaks are attributable to the stretching of the symmetrical and asymmetrical aryl rings, respectively [41,42].

**Figure 3:**
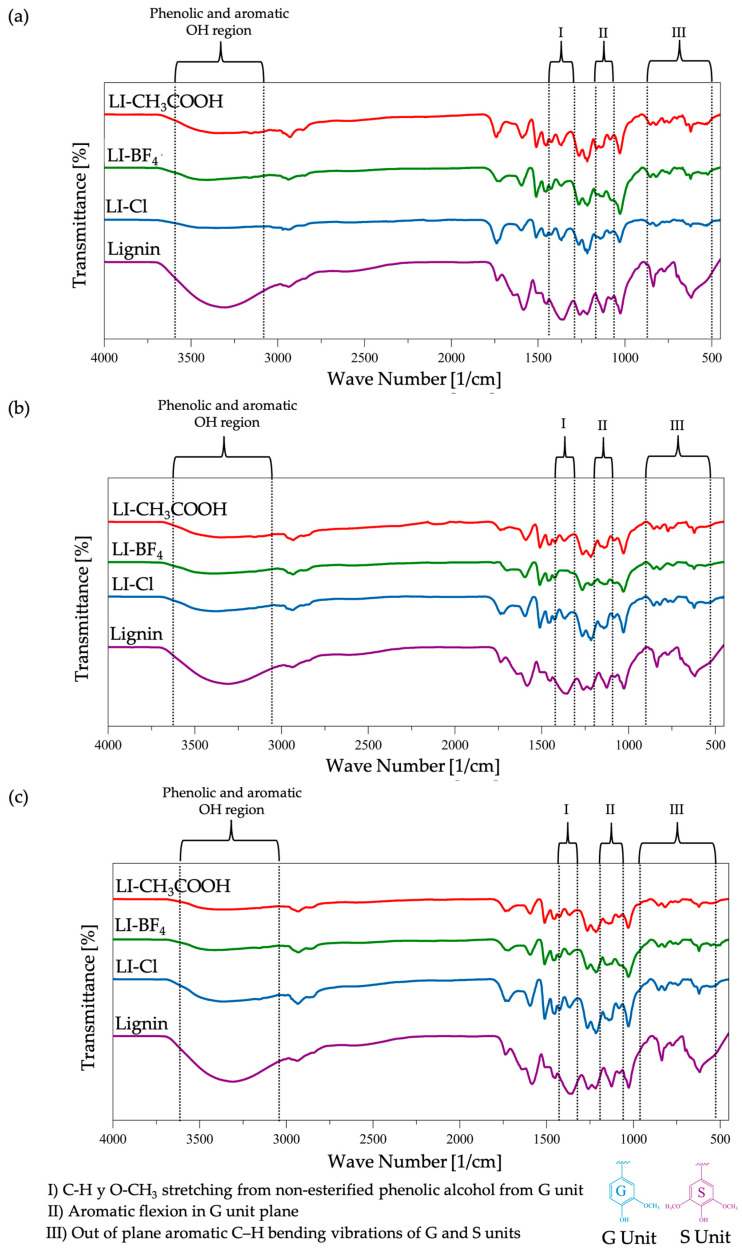
Infrared spectrums of the obtained lignin from acid depolymerization assisted by ionic liquids in the study with: (**a**) [EMIM^+^] cation, (**b**) [BMIM^+^] cation and (**c**) [HMIM^+^] cation.

Additionally, the 1461 and 1452 cm^−1^ peaks correspond to the asymmetric flexions of the C-H and C-C groups, respectively. The spectral region below 1400 cm^−1^ is more difficult to analyze because the majority of the bands are complex contributions of different vibrational modes; however, this region contains vibrations that are specific for the different monolignol units and permits structural characterization of lignin, as well as the peaks at 1336 and 1354 cm^−1^ that correspond to the asymmetrical flexions C-H and O-CH_3_ of the non-esterified phenolic alcohol of the guaiacyl unit; the peak at 1252 cm^−1^ corresponds to stretching of the guaiacyl ring, meanwhile the peak at 1216 cm^−1^ is attributed to the stretching of C-O, C-C and C=O, and the peak at 1126 cm^−1^ corresponds to the aromatic flexion of C-H in the same plane as the guaiacyl ring [7,43]. The peak at 1077 cm^−1^ corresponds to the stretching of C=O from aliphatic ether and the secondary alcohol; the peak at 1025 cm^−1^ corresponds to the vibrational mode C-O of the guaiacyl and syringyl ring; and lastly, the peaks at 877, 832 and 773 cm^−1^ correspond to the flexions outside of the plane of the aromatic rings of the guaiacyl and syringyl units; these results are consistent with the data reported in the bibliography, as well as the infrared spectroscopy signal assignment tables found in the literature that reference the structural characterization of lignin and its most representative model compounds [7,44,45]. Furthermore, based on the lignin spectrums of dry lignin obtained during acid depolymerization assisted by ionic liquids, and for a better comprehension of the data, the results were classified by the type of cation that formed a part of the used IL, in other words, the [EMIM^+^], [BMIM^+^] and [HMIM^+^] families, and the obtained spectrums for each family are presented in the Figure 3a, Figure 3b and Figure 3c, respectively.

For the three families of ILs, it is observed that they conserve along the spectrum certain characteristic signs similar to lignin, like peaks at 2973 and 2983 cm^−1^ that correspond to the C-H stretching of the methyl groups (CH_3_) and methylene (CH_2_), the peak at 2839 cm^−1^ that corresponds to the C-H stretching of the O-CH_3_ group, and the aromatic skeletal vibrations in combination with C-H stretching in the region between 1643 and 1452 cm^−1^ that corresponds with the characteristics of Kraft lignin. A decrease in the intensity of the ample band between 3500 and 3100 cm^−1^ is attributed to the OH stretching of the phenolic and aliphatic groups, which suggests that a certain quantity of fractions of aromatic phenolic compounds and aliphatic groups were detached from the molecular structure of lignin during the acid hydrolysis process assisted with ionic liquids in the study.

A decrease in signal intensity was also found for the characteristic peaks identifying the monolignol units guaiacyl and syringyl (G and S, respectively) in lignin, suggesting a significant modification of the lignin macromolecular structure. Firstly, a decrease in peak intensity was observed at 1366 cm^−1^ that corresponds to the asymmetric flexions of C-H and O-CH_3_ from the non-esterified phenolic alcohol of guaiacyl unit. Subsequently, the signal at 1126 cm^−1^ is attributed to the C-H aromatic flexion in the guaiacyl ring plane, and lastly, a decrease in signals at 877 and 773 cm^−1^ that correspond to the flexions outside the guaiacyl and syringyl aromatic rings’ plane was observed. Although, the weak peaks at 853 and 817 cm^−1^ that correspond to the flexions outside the plane of the aromatic rings from the units of guaiacyl and syringyl continue to evidence the presence of these units in the treated lignin, the decrease of the intensity of the signal in these regions in Figure 3a–c like I, II and III suggest that a determined quantity of fractions of the monolignols have been detached from the original molecular structure.

These spectroscopic changes indicate a degradation of the polymeric network into lower molecular weight fragments, which migrate to the soluble phase during IL-assisted acid hydrolysis. This behavior is largely attributed to the capacity of the ionic liquids studied to cleave the abundant and characteristic bonds within the biopolymer, such as the β-O-4 linkage. Additionally, these results suggest that the mechanism involved in β-O-4 bond rupture during the acid hydrolysis applied in this work is consistent with the Brønsted acid-catalyzed mechanism reported by Jia and Cox [28,45], where guaiacyl units are released during the hydrolysis of lignin model compounds. While these FT-IR results serve as strong qualitative indicators of structural degradation and bond cleavage, further quantitative studies of the liquid phase are required to determine the specific yield of aromatic monomers released during the process.

## 3. Materials and Methods

### 3.1. Synthesis and Chemical Characterization of Ionic Liquids

Three IL precursors were used: 1-ethyl-3-methylimidazolium chloride, [EMIM^+^][Cl^−^], 1-butyl-3methylimidazolium chloride, [BMIM^+^][Cl^−^], and 1-hexyl-3-methylimidazolium bromide [HBMIM^+^][Br^−^]. The IL [EMIM^+^][Cl^−^], with a 98.5% purity, was proportionated by Sigma-Aldrich. The IL [BMIM^+^][Cl^−^] was synthesized by alkylation of 1-methylimidazolium with 1-chlorobutane. In order to carry out the reaction, the system was brought to a temperature of 75 °C for 48 h with constant agitation and an inert nitrogen atmosphere. To remove the excess of 1-chlorobutane, the sample was washed with acetonitrile. The remnant of acetonitrile was removed via heating in a vacuum at 70 °C for 12 h. The sample was cooled at room temperature, and the product of interest obtained was IL [BMIM^+^][Cl^−^] [46,47,48]. For the synthesis of IL [HMIM^+^][Br^−^], equimolar quantities of 1-methylimidazole and 1-bromohexane reacted at a temperature of 75 °C for 24 h, with constant agitation and an inert nitrogen atmosphere. To remove the excess of 1-bromohexane, the sample was washed with acetonitrile. The remnant of acetonitrile was removed via heating in a vacuum at a temperature of 70 °C for 12 h. The sample was cooled at room temperature, and the resulting product obtained was IL [HMIM^+^][Br^−^] [49].

The ILs with the tetrafluoroborate anion, [EMIM^+^][BF_4_^−^], [BMIM^+^][BF_4_^−^], [HMIM^+^][BF_4_^−^], were synthesized using the reaction route via metathesis. In order to carry out the reaction, an equimolar quantity of sodium tetrafluoroborate, NaBF_4_, was added to the IL precursor in acetone as a reaction medium, and the mixture was agitated for 24 h at room temperature and filtered; then, the excess acetone was removed via heating in a vacuum [50,51]. To remove the excess salt in the halide sample, the system was dissolved in dichloromethane and dried over anhydrous magnesium sulphate, MgSO_4_. Following this, the excess was filtered, and the excess solvent was removed via heating in a vacuum [52,53,54]. Due to the reactivity of the ions, acetate and chloride, the ILs [EMIM^+^][CH_3_COOH^−^], [BMIM^+^][CH_3_COOH^−^], [HMIM^+^][CH_3_COOH^−^] and [HMIM^+^][Cl^−^] were synthesized via an ion exchange reaction via hydroxide intermediaries [55,56,57].

The NMR experiments of proton (^1^H) and carbon (^13^C) were done using D_2_O as a solvent at 25 °C in an Ascend 400 spectrometer from Consola Bruker Avance III with an intensity of magnetic field of 400 MHz, and the size of the sample was approximately 25 mg of IL dissolved in 0.55 mL of deuterated solvent. The experiments of infrared spectroscopy were done with an infrared spectrometer with transformed Fourier and total attenuated reflection (FTIR-ATR) Perkin Elmer Spectrum 100 at room temperature, with a spectral resolution of 4 cm^−1^, 16 scans and without previous conditioning of the sample.

### 3.2. Acid Hydrolysis of Lignin Assisted with ILs

**Figure 4:**
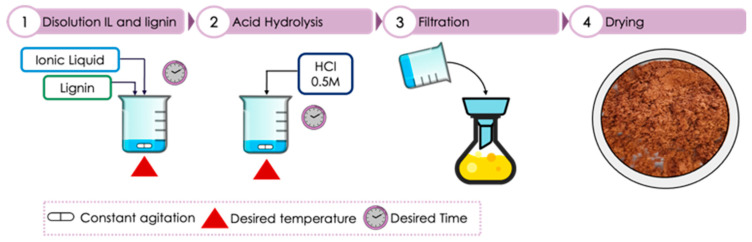
Schematic of ionic liquid-assisted acid hydrolysis of lignin.

The lignin used in the experimentation was commercial lignin alkali, which is a purified form of Kraft lignin from oak obtained in the Kraft pulp process. The alkali lignin was provided by Sigma-Aldrich. The methodology employed in the process of acid depolymerization assisted by the twelve ILs put forward is illustrated in Figure 4. As the first step, lignin was dissolved in the ionic liquid in an aqueous solution (70% weight in IL, 30% weight in water) at a ratio of lignin:IL 1:10. The aqueous IL was heated to 50 °C; subsequently, the desired amount of lignin was added and left in constant agitation for 30 min [44,58,59].

Once the lignin dissolved in IL, acid hydrolysis was executed, and two temperatures were taken into consideration, 80 °C and 110 °C; when the desired temperature was reached, 30 mL of HCl 0.5 M was added and left to react for 40 min under constant agitation in a controlled nitrogen atmosphere. After the reactions had concluded, distilled water was added to the mixture to stop the reaction, and the pH was leveled to neutral with a solution of NaOH 0.1 M, and to facilitate the separation of the phases, the mixture was cooled for 48 h. Subsequently, the mixture was mixed with the help of a vacuum pump, the solid residue was washed three times with distilled water to guarantee complete separation of IL from the solid phase, and then the solid residue was left to dry at a constant temperature of 70 °C for 24 h to completely ensure the elimination of water in the dry lignin. Once the lignin dried, the yields obtained from the reaction were obtained and characterized with spectroscopic techniques; the experiments were repeated three times to diminish the possibility of error in the results [60,61,62].

## 4. Conclusions

In this work, acid hydrolysis reactions were performed to evaluate the capacity to depolymerize the lignin molecules of a series of imidazolium-based ionic liquids. We aimed to observe the effect of the cation and anion on the capacity of the ionic liquid to depolymerize chains of two, four and six carbons, as well as the ions [Cl^−^], [BF_4_^−^] and [CH_3_COOH^−^], whose application in lignocellulosic biomass dissolution has previously been reported. The ratios of lignin:IL and IL:water used in the hydrolysis reactions were selected from bibliographical data. Once the acid hydrolysis reactions were concluded, the treated lignin was weighed and dried; the percentages of conversion obtained demonstrated the clear effect of both the anion and the cation on the capacity to depolymerize the ionic liquids used because it is observed that, with increasing chain length, there was a higher conversion percentage and vice versa. This is largely due to the capacity of the alkyl chains on the imidazolium rings to form stabilizing hydrogen bonds with the lignin polymer. Since the cation possesses electrostatic charges along the alkyl chain, a larger contact area increases the number of hydrogen bonds formed between the IL and lignin fractions. Furthermore, the IL’s efficiency in fractionation is enhanced by π–π stacking interactions between the imidazolium ring and the phenylpropane units of lignin.

Regarding the anion effect, lignin conversion percentages followed the order [Cl^−^] > [CH3COOH^−^] > [BF_4_^−^]. These results concur with previous reports suggesting that ILs with highly coordinated ions, such as [BF_4_^−^], possess a lower capacity to dissociate the predominant bonds in lignin. It is hypothesized that the higher conversion achieved with the [Cl^−^] anion is due to the steric hindrance presented by the [CH3COOH^−^] ion, which increases the difficulty of interacting with the lignin polymer and disrupting its natural stacking interactions. Additionally, the effect of temperature was evaluated at 80 °C and 110 °C; however, no significant impact on conversion percentages was found, nor were the observed trends regarding cation or anion effects altered by temperature.

The structural changes in lignin following acid hydrolysis were analyzed by infrared spectroscopy, revealing a decrease in signals corresponding to the OH stretching of phenolic and aliphatic groups, suggesting a reduction of these functional groups in the native structure. Furthermore, a decrease in the intensity of signals associated with the aromatic stretching and bending of guaiacyl and syringyl units suggests that these monolignol units were released during acid hydrolysis and are present in the liquid phase along with the IL and water. To accurately identify the phenolic fractions obtained, further characterization of the liquid phase using liquid chromatography (HPLC) and mass spectrometry (GC-MS) is required. Additionally, to track structural changes in the lignin molecule more precisely, quantitative spectroscopic techniques such as ^1^H, ^13^C, and ^31^P NMR should be employed. Nevertheless, the results of this study provide valuable insights for the design of ILs capable of depolymerizing lignin into low molecular weight fractions.

## Figures and Tables

**Table 1:** Proposed ionic liquids.

Ionic Liquids			
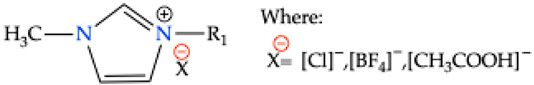			
R1	Anion (X–)	Name	Abbreviation
C_2_H_5_	[CH_3_COOH^−^]	1-ethyl-3-methylimidazolium acetate	[EMIM^+^][CH_3_COOH^−^]
C_4_H_10_	[CH_3_COOH^−^]	1-Butyl-3-methylimidazolium acetate	[BMIM^+^][CH_3_COOH^−^]
C_6_H_14_	[CH_3_COOH^−^]	1-hexyl-3-methylimidazolium acetate	[HMIM^+^][CH_3_COOH^−^]
C_2_H_5_	[Cl^−^]	1-ethyl-3-methylimidazolium chloride	[EMIM^+^][Cl^−^]
C_4_H_10_	[Cl^−^]	1-Butyl-3-methylimidazolium chloride	[BMIM^+^][Cl^−^]
C_6_H_14_	[Cl^−^]	1-hexyl-3-methylimidazolium chloride	[HMIM^+^][Cl^−^]
C_2_H_5_	[BF_4_^−^]	1-methyl-3-methylimidazolium tetrafluoroborate	[EMIM^+^][BF_4_^−^]
C_4_H_10_	[BF_4_^−^]	1-hexyl-3-methylimidazolium tetrafluoroborate	[BMIM^+^][BF_4_^−^]
C_6_H_14_	[BF_4_^−^]	1-hexyl-3-methylimidazolium tetrafluoroborate	[HMIM^+^][BF_4_^−^]

**Table 2:** Percentages of conversion of lignin obtained during acid depolymerization assisted with ionic liquids at different temperatures.

Ionic Liquid	% of Conversion	
	T = 80 °C	T = 110 °C
[EMIM^+^][Cl^−^]	30.02	31.52
[EMIM^+^][BF_4_^−^]	11.84	16.57
[EMIM^+^][CH_3_COOH^−^]	26.23	30.16
[BMIM^+^][Cl^−^]	41.2	42.43
[BMIM^+^][BF_4_^−^]	35.81	37.95
[BMIM^+^][CH_3_COOH^−^]	36.49	38.67
[HMIM^+^][Cl^−^]	47.81	49.24
[HMIM^+^][BF_4_^−^]	36.10	37.90
[HMIM^+^][CH_3_COOH^−^]	37.65	39.15

## Data Availability

The data presented in this study are available upon request from the corresponding author.
